# Bridging mental health, cognition and the brain in mild traumatic brain injury: A multilayer network analysis of the TRACK-TBI study^☆^

**DOI:** 10.1016/j.nicl.2026.103957

**Published:** 2026-02-02

**Authors:** Juan F. Domínguez D., Mervyn Singh, Lyndon Firman-Sadler, Jade Guarnera, Ivan L. Simpson-Kent, Phoebe Imms, Andrei Irimia, Karen Caeyenberghs, the TRACK-TBI Investigators

**Affiliations:** a Cognitive Neuroscience Unit, School of Psychology, Deakin University, Melbourne, VIC, Australia; b Child and Adolescent Imaging Research (CAIR) Program, University of Calgary, Calgary, Alberta T2N 1N4, Canada; c Alberta Children’s Hospital Research Institute, University of Calgary, Calgary, Alberta T2N 1N4, Canada; d Hotchkiss Brain Institute, University of Calgary, Calgary, Alberta T2N 1N4, Canada; e Department of Radiology, Cumming School of Medicine, Calgary, Alberta T2N 1N4, Canada; f Institute of Psychology, Developmental and Educational Psychology Unit, Leiden University, Leiden, the Netherlands; g School of Biomedical Sciences, the University of Queensland, Brisbane, Queensland; h QIMR Berghofer Medical Research Institute, Herston, QLD, Australia; i Ethel Percy Andrus Gerontology Centre, Leonard Davis School of Gerontology, University of Southern California, Los Angeles, CA, USA; j Department of Biomedical Engineering, Viterbi School of Engineering, University of Southern California, Los Angeles, CA, USA; k Department of Quantitative & Computational Biology, Dana and David Dornsife College of Arts & Sciences, University of Southern California, Los Angeles, CA, USA

**Keywords:** Multilayer Network Analysis, Cognition, Mental-health, Brain structure, Traumatic Brain Injury

## Abstract

**Background:**

People with mild traumatic brain injury (mTBI) suffer from several mental health symptoms (*e.g.*, anxiety, depressive symptoms) and cognitive deficits (*e.g.*, attentional deficits, slowed processing speed). However, symptoms in TBI are largely investigated in isolation, using univariate approaches, ignoring interactions between symptoms and the underlying large-scale brain networks. We constructed the first multilayer network in mTBI to examine relationships between networks of cognition, mental health and structural brain measures and to identify key variables bridging relationships across these networks.

**Methods:**

Chronic phase cross-sectional data (6-month follow-up) from 457 mTBI participants was extracted from the TRACK-TBI Longitudinal study. We selected four variables from self-report mental health questionnaires (affective layer), eight cognitive test scores from the NIH toolbox (cognitive layer), and gray matter volumes from eight brain regions of the central executive and salience networks from anatomical MRI scans (brain layer). We used a multilayer network approach to examine the relationships (edges) between all variables (nodes) across layers. We then used the bridge strength centrality metric to identify nodes that ‘bridge’ the affective, cognitive, and brain layers.

**Results:**

In this sample of mTBI participants, across all affective and cognitive layer nodes, only impairments in insomnia were noted. Multilayer network analysis revealed insomnia severity, immediate verbal memory, somatisation and processing speed nodes exceeded an *a priori* 80th percentile threshold on the bridge strength scores and may therefore be regarded as key nodes potentially bridging relationships across affective, cognitive and brain layers.

**Conclusions:**

The bridging nodes identified in our multilayer network analyses may suggest targets for future studies to develop more customized, efficient, and efficacious treatments to alleviate mental health symptoms and cognitive deficits in mTBI.

## Highlights

• Multilayer-network analysis allows investigation of brain-behaviour relationships within the same framework.
• Bridge strength centrality can identify variables that ‘bridge’ the brain and behavioural domains in such networks.
• Insomnia severity, immediate verbal memory, somatisation and processing speed are bridges in mild traumatic brain injury.
• These bridges can in the future help pinpoint potential training targets and strategies to improve outcomes in this cohort.

## 1 Introduction

In 2019, there were approximately 12.3 million incident cases of mild traumatic injury (mTBI) worldwide (Wu et al., 2025). Between 20–40% of people with mTBI experience long-term deficits (Carroll et al., 2020). A number of studies have reported reduced performance in people with mTBI on cognitive tasks assessing attention, memory, attention switching, and executive functioning (Barman et al., 2016, Hacker et al., 2023, Imms et al., 2024, Miotto et al., 2010, Tsai et al., 2021), together with increased mental health symptoms (depression, anxiety, stress) (Howlett et al., 2022, Karakurt et al., 2021), that present long-term (>3 months) post injury. Moreover, studies have demonstrated bidirectional relationships between cognitive function and mental health in TBI (Keatley et al., 2023, Uiterwijk et al., 2022). These chronic impairments are considered the result of disruptions in the coordinated activity of three neural networks (*i.e.*, the default mode network, salience network, and central executive network (Amgalan et al., 2022, Li et al., 2023, Liu et al., 2024, Menon, 2011, van der Horn et al., 2023).

There is mounting evidence from network psychometrics that mental health issues result from the interaction between multiple symptoms in a symptom network (*e.g.*, depressed mood, fatigue, insomnia, difficulty in concentration, motivation), with disruption to a symptom spreading across the network (Borsboom et al., 2017, Carmichael et al., 2023, Schubert and Frischkorn, 2020, van der Maas et al., 2021, Yang et al., 2022). In the context of moderate-severe TBI, Carmichael and colleagues (Carmichael et al., 2023) showed that the symptoms of worrying and having difficulty relaxing are especially important to the maintenance and comorbidity of post-TBI anxiety and depression. Despite the growing recognition of symptoms as network phenomena, when attempting to ascertain how disruption to the brain networks leads to specific symptoms, studies have focused on isolated behavioural measures that are not in the same statistical framework as the brain measures. It is, however, important to analyse brain-behaviour relationships using the same framework (Tiego and Fornito, 2023).

Multilayer network analysis is a new approach that allows analysis of brain-behaviour relationships within the same framework: in one implementation, the relationships among variables across levels of organisation, such as behaviour/symptoms and brain variables are modelled simultaneously by placing them all in the same integrated network (Blanken et al., 2021). This allows for the concurrent visualization and understanding of a set of complex relationships between variables across levels of organization. Variables are represented as nodes within multiple layers of this network and their relationships can be modelled as partial correlations between nodes within the same layer and across different layers. This has the added benefit that we can be more confident that the observed relationships are true, as the confounding effects of all other variables in the network are controlled for. Centrality metrics (*e.g.*, bridge strength centrality) are then used to identify nodes that ‘bridge’ the brain and behavioural layers. These bridge nodes exhibit particularly strong relationships with nodes from layers different from their own. More specifically, bridge nodes can be regarded as nodes that mediate relationships between different layers (brain and symptom layers in the present case), so that changes (*e.g.*, worsening of symptoms, brain tissue loss) in such nodes have a higher likelihood of spreading to other nodes across layers (Borsboom, 2017, Jones et al., 2019). Bridge layers may thus serve as previously unrecognized targets for intervention. Further, the approach can also establish a hierarchy of edges and identify important interlayer edges, which can reveal likely paths for the spread of changes across network layers. Multilayer network analysis is still in its infancy and, to our knowledge, only three other studies have used this approach to study brain-psychometric relationships, in depression (Hilland et al., 2020), autism (Bathelt et al., 2022), and intelligence in struggling learners (Simpson-Kent et al., 2021).

In the present study, we employed, for the first time, this innovative multilayer integrated network approach to examine the relationships between networks of mental health measures, cognitive scores, and gray matter brain volumes (GMV) in chronic mTBI, using a subset of the Transforming Research and Clinical Knowledge in TBI Longitudinal (TRACK-TBI LONG) dataset (Bryant et al., 2023, Palacios et al., 2022, Sibilia et al., 2022). Our aim was to characterize the complex interactions between behavioural symptoms and underlying brain mechanisms in mTBI. More specifically, we first wanted to apply a network psychometrics approach to model mental health (*e.g.*, depression, anxiety) and cognitive functioning (*e.g.*, processing speed, memory) as a system of interacting symptoms. The specific set of measures used in this paper are part of the TBI Common Data Elements and validated as part of the TRACK-TBI study (Yue et al., 2013). They were selected because they have been previously reported to be affected in TBI and are concomitant or even correlated with each other in this clinical population (Keatley et al., 2023, Montgomery et al., 2022, Polinder et al., 2018, Silver et al., 2009, Silverberg and Iverson, 2011, Stenberg et al., 2020, Stubbs et al., 2020, Uiterwijk et al., 2022 Wickwire et al., 2018). Given the likely causal role of mental health symptoms on cognitive function in TBI (Keatley et al., 2023, Silver et al., 2009, Silverberg and Iverson, 2011, Uiterwijk et al., 2022), we hypothesised that the mental health nodes would act as bridging nodes in mTBI.

Second, we wanted to simultaneously model brain-behaviour relationships by integrating the behavioural symptom network (comprised of the cognitive functioning and mental health layers) with the brain network. GMVs were selected from regions of the salience and the executive control subnetworks, given their direct involvement in cognitive and mental health deficits across a range of neurological and psychiatric disorders, including TBI (Li et al., 2023, Liu et al., 2024, Menon, 2019, Menon, 2011, Menon and D'Esposito, 2022, van der Horn et al., 2023). Our aim was exploratory and consisted primarily in identifying bridging nodes between the mental health, cognitive functioning and GMV networks in mTBI.

## 2 Methods

### 2.1 Participants and study design

This cohort study used data for 457 mTBI participants drawn from the TRACK-TBI LONG project, a multicentre cohort study conducted across 18 Level 1 trauma centres in the US (Bryant et al., 2023, Palacios et al., 2022, Sibilia et al., 2022, Yue et al., 2013). Eligible mTBI participants were included if they were: (1) aged 16 years and older; (2) presented with a documented case of TBI within 24 h after injury; (3) obtained a clinical computed tomography (CT) scan within 24 h after injury; (4) exhibited a disorder of consciousness, which refers to loss of consciousness, loss of memory and alterations of mental state (*e.g.*, feeling dazed, disoriented, confused), as per American Congress of Rehabilitation Medicine (ACRM) Criteria for mild TBI (Kay et al., 1993); and (5) reported a total Glasgow Coma Scale (GCS) score of 13–15 points (indicating presence of mild TBI) upon admission. Participants were excluded if they were incarcerated, pregnant, or diagnosed with any of the following: (1) penetrating TBI, (2) psychiatric disorders, (3) neurological disorders; and (3) non-English-speaking. Written consent was granted by all enrolled participants and/or their legally authorised representatives prior to participation in the study. Enrolled participants completed a battery of behavioural and self-report outcome assessments and underwent multimodal MRI scanning within each participating institution. Full details of all outcome measurements used in TRACK-TBI LONG can be accessed via the Standard Operating Procedures for Outcome Assessment Manual (https://tracktbi.ucsf.edu/researchers). For this study, we extracted secondary data from the 6-month follow-up time point. Participants with mTBI were only included in the final sample if they had *complete* behavioural and neuroimaging data.

### 2.2 Mental health measures

Participants completed the *BRIEF Symptom Inventory 18 (BSI-18)* and the *Insomnia Severity Index (ISI)*. From these questionnaires, the following measures of symptom severity were selected as nodes for our mental health layer: (1) Anxiety (ANX), (2) Depression (DEP), (3) Somatization (SOM), and Insomnia (INSOM). The Global Severity Index (GSI) score, calculated by summing all ANX, DEP and SOM scores, was also obtained for sample characterization. See the Supplementary Material for a full description.

### 2.3 Cognitive measures

A core battery of neuropsychological tests from the TRACK-TBI LONG Comprehensive Assessment Battery (https://tracktbi.ucsf.edu/researchers) was also administered. The following measures were selected as nodes for our cognitive layer: (1) Processing speed index (ProcS*)* (from Symbol Search and Coding subtests of the Wechsler Adult Intelligence Scale IV), (2) psychomotor speed (PsyS) and cognitive flexibility (CogFl) [from the Trail Making Test Part A (TMTA) and Part B (TMTB)], and (3) immediate (vIMM), interference (vINTER), and delayed verbal recall (vDELAY) [from the Rey Auditory Verbal Learning Test II (RAVLT-II)]. See the Supplementary Material for a full description.

### 2.4 Neuroimaging

#### 2.4.1 MRI data Acquisition

We used the T_1_-weighted anatomical MRI data obtained 6 months post-injury. MRI scanning was performed on 3 T scanners across all sites. Scanning sequences have been previously described (Sibilia et al., 2022).

#### 2.4.2 Gray matter volumes

**Fig. 1:**
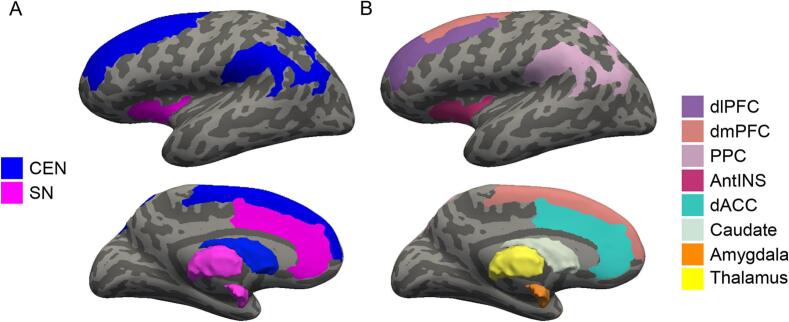
Regions of interest. (A) Visual representation of the Central Executive (CEN) and Salience (SN) networks. (B) Individual cortical and subcortical CEN and SN gray matter regions. Note: dlPFC: Dorsolateral Prefrontal Cortex; dmPFC: Dorsomedial Prefrontal Cortex; PPC: Posterior Parietal Cortex; CAU: Caudate; AntINS: Anterior Insula; dACC: Dorsal Anterior Cingulate Cortex; AMYG: Amygdala; THAL: Thalamus.

FreeSurfer (6.0) (Fischl, 2012) was used to obtain cortical and subcortical regions of interests (ROI) using the Destrieux atlas (Destrieux et al., 2010, Fischl et al., 2004). GMV was calculated for each ROI (bilateral average). The Total Intracranial Volume (TIV) was also estimated in FreeSurfer using an atlas scaling factor derived from the registration to an average template using an affine transformation (Buckner et al., 2004). We selected gray-matter ROIs corresponding to the Central Executive (CEN) and Salience (SN) systems (Li et al., 2023, Liu et al., 2024, Menon, 2011, van der Horn et al., 2023). See Fig. 1 for a visual representation. ROIs from CEN included: (1) Dorsolateral Prefrontal Cortex (dlPFC), (2) Dorsomedial Prefrontal Cortex (dmPFC), (3) Posterior Parietal Cortex (PPC), and (4) Caudate (CAU). ROIs from SN included: (1) Anterior Insula (AntINS), (2) Dorsal Anterior Cingulate Cortex (dACC), (3) Amygdala (AMYG); and (4) Thalamus (THAL). A full list of gray matter parcellations used to create these ROIs is presented in Supplementary Table S1.

### 2.5 Statistical procedures

#### 2.5.1 Network estimation

**Fig. 2:**
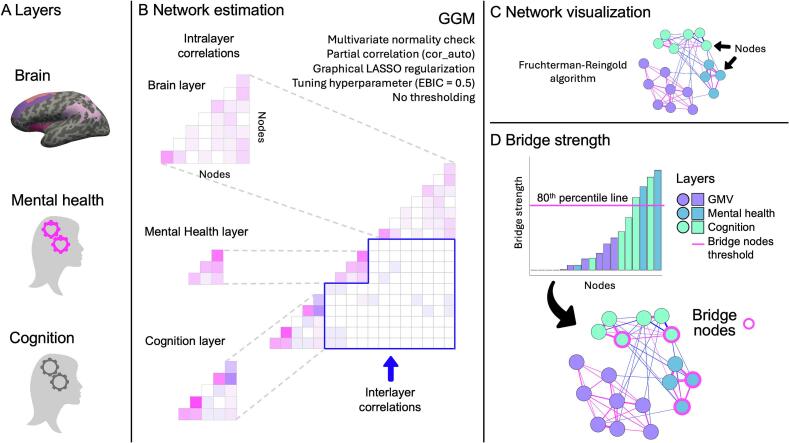
Workflow to estimate multilayer networks and bridge strength. (A) Network layers selection; (B) Multilayer network estimation with Gaussian Graphical Models (GGM) using LASSO regularisation (a tri-layer network is shown in the example); (C) Network visualization with the *Fruchterman-Reingold algorithm*; (D) Bridge strength computation. Centrality values for each node are ranked in ascending order. Bridging nodes are ascertained using an 80th percentile threshold on the raw scores of bridge strength. The bridge nodes are highlighted in the network visualization (magenta outline). See text for full details. (For interpretation of the references to colour in this figure legend, the reader is referred to the web version of this article.)

**Fig. 3:**
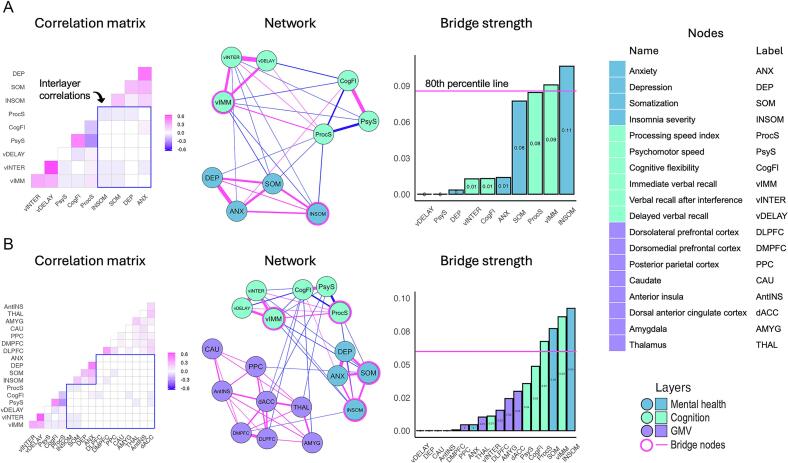
Results of multilayer network analysis in mTBI. (A) Mental health-cognition bi-layer. (B) Mental health-cognition-GMV layer. Note: Weights between −0.05 and 0.05 have been set to ± 0.05 in the partial correlation matrices to increase their visibility.

Network estimation was conducted in R (4.4.1, “Race for Your Life”; R Core Team, 2021) using the *bootnet* (1.6) (Epskamp et al., 2018a), *igraph* (2.0.3) (Csárdi et al., 2006), and *networktools* (1.5.2) (Jones, 2017) packages. Fig. 2 illustrates the workflow to estimate our networks. Nodes included in the networks measured mental health outcomes (4 nodes: ANX, DEP, SOM, ISI), cognitive functioning (6 nodes: ProcS, PsyS, CogFl vIMM, vINTER, vDELAY), and GMV in regions of the CEN and SN (8 nodes – 4 for CEN: dlPFC, dmPFC, PPC, CAU; 4 for SN: AntINS, dACC, AMYG, THAL) (see Fig. 3 for a list of nodes and labels used throughout). We constructed a bi-layer network with mental health and cognition layers and a tri-layer network with the mental health, cognition and GMV layers. Residualization was used to remove the effect of age from all nodes, and the effect of total intracranial volume (TIV) and site from GMV nodes, prior to network and bridge centrality estimation (Bethlehem et al., 2022, Storsve et al., 2014).

Relationships between nodes (*i.e.*, edges) were estimated as undirected, weighted partial correlations with Gaussian Graphical Models (GGM) using graphical *LASSO* regularisation (Friedman et al., 2007). Following Simpson-Kent and colleagues (2021), we chose LASSO as it generates sparser networks at the expense of more complex models. As such, LASSO decreases the risk of potentially spurious (that is, false positive) correlations, generates simpler visualizations and makes the conditional dependencies between nodes more straightforwardly interpretable (Epskamp et al., 2018a, Epskamp et al., 2018b). Due to violations of multivariate normality, edge weights for all our networks were estimated using the “cor_auto” function, which automatically computes the correct type of correlation (*e.g.*, Pearson and/or Spearman) for the data. Network density was determined using the Extended Bayesian Information Criterion (EBIC), with the regularisation tuning parameter γ set to 0.5 to control the sparsity of the network. We considered a network density (actual/possible edges) of 50% or more to be high density; 30% to 50% as medium density, and < 30% as low density (Carmichael et al., 2023). Topological characteristics of each network were visualised in *qgraph* (1.6.5) (Epskamp et al., 2012), with nodes depicted as circles and edges as lines connecting the nodes. Visualisation of all networks followed the *Fruchterman-Reingold algorithm* (Fruchterman and Reingold, 1991).

#### 2.5.2 Bridge strength

*Bridge strength centrality* between a set of communities (or layers) that were manually prespecified (based on their layer, *i.e.*, mental health, cognition, or GMV) was then estimated to identify nodes that bridge the different layers. Bridge strength indicates a node’s total connectivity with layers other than its own. It is computed as the sum of the *absolute value* of every edge weight that connects a node in one community with a node in another community (Jones et al., 2019). Centrality values for each node were then ranked in ascending order. Nodes were designated as bridging nodes by using an 80th percentile threshold on the raw scores of bridge strength (Jones et al., 2019).

The stability of bridge strength was assessed by calculating the correlation stability (CS) coefficient between the bridge strength calculated from the initial sample, and estimates calculated after a proportion of participants have been dropped (over 2000 bootstraps). CS coefficients denote the estimated maximum number of individuals that can be dropped to retain, with 95% probability, a correlation of at least 0.70 between the two samples. CS coefficients ≥0.50 suggest that the centrality measures are stable and are unlikely to be influenced by variability in sample size (Epskamp et al., 2018a). For CS coefficients between 0.25 and 0.5, centrality measures should be interpreted with caution; if CS falls below 0.25, centrality measures are not stable and should not be interpreted.

It is important to highlight that the 0.5 and 0.25 cutoffs, as originally formulated by Epskamp et al. (2018a), are somewhat arbitrary and should not be taken as definite guidelines. These cutoffs were, in addition, set for psychometric networks. Therefore, it remains an open question whether they need to be as stringent with non-psychological data (*e.g.*, brain measures, as in the present manuscript). Moreover, the simulations conducted in the Epskamp et al. (2018a) paper to determine these cutoffs were evaluated with reference to single layer centrality indices (betweenness, closeness and strength) in psychological networks, not bridge centrality indices (*e.g.*, bridge strength) in multilayer networks.

## 3 Results

### 3.1 Demographic and clinical characteristics

Table 1 (next page) presents all the demographic and clinical characteristics of our mTBI sample, as well as their scores in the mental health and cognitive measures. For a full characterization of the mTBI sample, we compared their demographic, clinical and GMV data to an orthopaedic trauma injury control group. The control group was recruited as part of the TRACK-TBI study and was comprised of 86 participants aged 20–71 years (32 females; M (age) = 38.49 years; SD (age) = ±14.88 years). Independent samples T-tests (corrected for multiple comparisons using the False Discovery Rate at p = 0.05) revealed that mTBI patients had significantly higher severity of insomnia symptoms compared to Controls (*p* = 0.02). Of relevance, a total of 192 or 42% of mTBI participants reported having experienced either subthreshold (n = 112), moderate (n = 60) or severe (n = 20) symptoms of insomnia. No significant group differences were found for any of the other mental-health or cognitive outcome measures (see Supplementary Table S3). Regression analysis (correcting for the effect of TIV and site) failed to detect statistically significant group differences between TBI patients and Controls in GMV across all ROIs (see Supplementary Table S4).

**Table 1:** Demographic and Clinical Characteristics.

	**mTBI (n = 457, 150 females)**			
	M	SD	Min	Max
Age	38.33	15.98	17	83
Days since baseline	182.11	7.98	162	200
Anxiety (BSI-18)	2.74	4.19	0	23
Somatization (BSI-18)	2.54	3.71	0	19
Depression (BSI-18)	2.83	4.37	0	21
GSI (BSI-18)	8.11	11.02	0	61
Total Insomnia Severity (ISI)	7.53	6.92	0	28
Processing speed Index (WAIS-IV PSI)	102.07	15.07	53	150
Psychomotor speed (TMTA)	25.70	10.85	10.47	86
Cognitive flexibility (TMTB)	68.17	39.64	25	330
Immediate verbal recall	48.09	10.67	5	72
Verbal recall interference	9.93	3.27	1	15
Delayed verbal recall	9.70	3.49	0	15

*Note:* mTBI: mild Traumatic Brain Injury; BSI-18: Brief Symptom Inventory 18; ISI: Insomnia Severity Index; WAIS-IV PSI: Processing Speed Index of the WAIS-IV; TMTA: Trail Making Test Part A; TMTB: Trail Making Test Part B.

### 3.2 Network analysis

Descriptive statistics for all multilayer networks in mTBI participants are presented in Table 2 (next page) and results of the multilayer network analysis (including the regularised partial correlation matrices) are graphically represented in Fig. 3.

**Table 2:** Descriptive statistics and stability measures for the Psychometric bi-layer network and the Integrated Brain-Psychometric tri-layer network in mTBI participants.

	Edge weight mean (SD), [range]	Non-zero edges/Total No. edges (density)	Bridge Strength stability (CS†)
**Mental health-cognition** **bi-layer network**	0.06(0.18), [-0.32 0.65]	28 / 45 (62.2%)	0.27
**Mental health-cognition-GMV tri-layer network**	0.03(0.10), [-0.30 0.60]	53 / 153 (34.6%)	0.28

† CS, correlation stability.

#### 3.2.1 Mental health-cognition bi-layer network of mTBI participants

The density of the mental health-cognition network was high, with 62.2% of possible edges present. This network showed positive and negative partial correlations, with edge weights varying between small and high values (see Table 2). The strongest (positive) edges were between nodes within the same layer, *e.g.*, vINTER-vDELAY (0.65), DEP-ANX (0.53), and PsyS-CogFl (0.49). Interlayer edges were mostly negative, including ProcS-INSOM (−0.07), SOM-vIMM (−0.05), INSOM-vIMM (−0.04), ProcS-SOM (−0.02), vINTER-SOM (−0.01), and ANX-CogFl (0.01). Regarding bridge centrality, two nodes emerged as bridge nodes in the mental health-cognition bi-layer network, with INSOM having the highest bridge strength, followed by vIMM. The stability of these centrality estimates was CS = 0.27.

#### 3.2.2 Mental health-cognition-GMV tri-layer network in mTBI participants

The density of the health-cognition-GMV network was medium-to-low, as 34.6% of possible edges were present. It had both positive and negative partial correlations, with edge weights ranging between small and high values (see Table 2). Consistent with the bi-layer network, the strongest edges were within-layer, predominantly positive, and included the edges vINTER-vDELAY (0.60), DEP-ANX (0.50), PsyS-CogFl (0.46), and DLPFC-DMPFC (0.39). In the mental health-cognition-GMV tri-layer network, interlayer the observed edges included ProcS-INSOM (−0.05), vIMM-SOM (−0.05), vIMM-INSOM (−0.04), PsyS-dACC (−0.03), CogFl-AMYGD (−0.02), CogFl-DLPFC (−0.02), vINTER-SOM (−0.01), and PsyS-THAL (−0.01) (a number of additional interlayer edges were observed that had edge weights below an absolute strength of 0.01, including INSOM-DMPFC, SOM-PCC and others). Four nodes could be identified as bridge nodes. These included (in descending order): INSOM, vIMM, SOM, and ProcS. Regarding stability, the CS coefficient was 0.28.

## 4 Discussion

In the present study, we constructed for the first time multilayer networks in mTBI participants that integrated self-report questionnaires of mental health, cognitive tests scores from the NIH toolbox, and GMV from anatomical MRI scans. We found four nodes passed the centrality threshold in our multilayer networks, including *insomnia severity*, *immediate verbal recall*, *somatisation*, and *processing speed*. In the next sections, we will discuss these bridging nodes, the clinical implications, limitations of the study and possible avenues for future research.

### 4.1 Mental health-cognition bi-layer network in mTBI participants

The mental health-cognition network in mTBI participants had a high density. This finding adds to evidence showing that TBI leads to a constellation of interrelated sequelae involving both affective and cognitive symptoms (Keatley et al., 2023, Silver et al., 2009, Silverberg and Iverson, 2011, Uiterwijk et al., 2022). This is in agreement with Carmichael and colleagues (Carmichael et al., 2023), who found that their network of anxiety and depressive symptoms in individuals with moderate-to-severe TBI had medium density, which confirms the comorbidity and reciprocity of anxiety and depression in this population (Gould et al., 2011, Wang et al., 2021). Our present findings further show that this is the case not only for anxiety and depression, but other affective symptoms and cognitive deficits as well. This aligns with the view of network psychometrics according to which the effect of a symptom is more likely to spread throughout a dense, strongly interconnected symptom network, potentially leading to a self-sustaining, ingrained mental health disorder (Borsboom, 2017). However, it is important to highlight that, while network psychometrics (and more specifically the network theory of mental disorders; Borsboom, 2017) posits causal connections between symptoms though a myriad of biological, psychological and societal mechanisms, we cannot assert actual causal relationships between the specific symptoms we chose for this study, given the correlational nature of our analysis. Moreover, as there may be substantial overlap between nodes in our network (*e.g.*, depression and anxiety), it may not be that one is causing the other, but that they reflect a single syndrome or disruption of a common underlying circuit.

*Insomnia severity* and *immediate verbal recall* were found to be central nodes that bridge the mental health and cognitive layers. Therefore, our hypothesis that the mental health nodes would act as bridging nodes in the mental health-cognition bi-layer network in mTBI was only partially supported in that the node with the highest bridge strength (*insomnia severity*) was a mental health node, but the other bridging node (*immediate verbal recall*) was a cognitive node. Studies have demonstrated that alterations in sleep quality and quantity are commonly found after mTBI (Montgomery et al., 2022, Wickwire et al., 2018). Approximately 50% of people with mTBI report insufficient and disturbed sleep. We similarly found that 42% of our sample experienced either subthreshold, moderate or severe symptoms of insomnia. Moreover, *insomnia severity* was the only psychometric measure where the mTBI sample significantly differed from controls. We also found that *insomnia severity* had strong negative edges with cognitive nodes, including *immediate verbal recall* (the other bridge node in this network) and *processing speed index* (the strongest interlayer edge). This is noteworthy, as *insomnia severity* was the bridge node with the highest bridge strength in both the bi- and tri-layer networks (see section 4.2 below). People with mTBI often experience deficits in *immediate verbal recall* and have a shorter verbal memory span, making it harder to recall a list of words or numbers immediately after hearing them (Vakil et al., 2019). Tests used to estimate episodic memory abilities (Rabin et al., 2005) have revealed high levels of sensitivity and specificity to disorders causing verbal memory dysfunction, including TBI (Kennedy et al., 2024).

Our results align with clinical studies showing that sleep problems post-injury are associated with greater cognitive impairment in mTBI (Kalmbach et al., 2018, Werner et al., 2021). Future research should continue to examine the relationships between cognitive performance and mental health outcomes after TBI, rather than explore them in isolation. Understanding the interplay between these symptoms is also essential for comprehensive care of people with TBI (Vakil et al., 2019). Effective rehabilitation programs addressing verbal memory may result in improvements in mental health.

### 4.2 Mental health-cognition-GMV tri-layer network in mTBI participants

Bridge centrality analysis revealed the existence of four nodes that served to bridge layers: *insomnia s*everity and *immediate verbal recall (*which were the same as in the mental health-cognitive bi-layer network; see discussion above in section 4.1), as well as *somatization* and *processing speed index.* All four bridge nodes were interconnected, reaffirming their centrality in the multilayer network. Our results highlight the importance of mental health and cognitive functioning in understanding brain-behaviour relationships in mTBI.

As noted earlier, *insomnia severity* was the bridge node with the highest bridge strength. In the context of the tri-layer network, it is important to highlight that the GMV layer node *insomnia severity* was connected to is DMPFC. These nodes were connected via a negative edge, indicating that decrease in DMPFC volume was associated with greater insomnia severity symptoms. This is noteworthy as there is a well-known association between sleep regulation and DMPFC. The DMPFC is a critical node in the arousal network that controls the level of consciousness (Mashour et al., 2022). In addition, using magnetoencephalography, Ioannides and colleagues (2009), reported that activity in DMPFC increased the most during both rapid eye movement (REM) and deep non-REM sleep. Age-related atrophy of this structure has been linked to reduced non-REM slow wave activity (Mander et al., 2013). Patients with persistent insomnia symptoms have been reported to exhibit cortical thinning in the DMPFC. Most relevant is that, in a sample of 192 TBI patients (veterans with penetrating brain injuries), damage to DMPFC was found to be associated with insomnia (Koenigs et al., 2010).

Regarding *somatization* (from the mental health layer)*,* previous work indicates that individuals with TBI, especially those with mTBI, often report somatic symptoms that are not directly attributable to the injury itself, such as gastrointestinal upset, headaches, dizziness, musculoskeletal pain, and cardiorespiratory complaints (Stenberg et al., 2020). These symptoms can persist long after the initial injury and can complicate recovery (Stubbs et al., 2020). *Processing speed index* (from the cognitive layer), which measures perceptual processing speed ability, has been shown to be affected across (complicated) mild, moderate and severe TBI (Carlozzi et al., 2015, Donders and Strong, 2014). Slower cognitive processing is well established in mTBI (Frencham et al., 2005, Hacker et al., 2023). For example, in their *meta*-analysis, Frencham and colleagues (Frencham et al., 2005) found significant deficits in neuropsychological measures, with information-processing speed showing the largest effect.

We observed that *somatization* had strong negative edges with cognitive nodes, including *immediate verbal recall* and *processing speed index* (both of which were bridging nodes), as well as *verbal recall interference*. Studies have demonstrated that the presence of somatic symptoms can exacerbate cognitive deficits, including processing speed (Calvillo and Irimia, 2020). Conversely, cognitive deficits can increase psychological distress, potentially leading to more pronounced somatic symptoms. Both somatization and processing speed deficits can significantly hinder the recovery process in TBI. Understanding and addressing this relationship is crucial for developing effective rehabilitation strategies for individuals with TBI.

While out of the four bridge nodes identified, only *insomnia severity* was found to be impaired in the mTBI sample, all four of them were interconnected by the strongest interlayer edges, including *processing speed* − *insomnia severity*, *immediate verbal recall* − *somatization*, and *immediate verbal recall – insomnia severity.* This suggests these nodes may act as a bridge cluster in the spread of injury effects across brain and behaviour networks (Borsboom, 2017, Jones et al., 2019), particularly in more severe TBI presentations, where we may find all four bridge nodes being affected following injury. Future research with individuals with more severe TBI is required to test this hypothesis. In addition, this bridge cluster may enhance the efficacy of interventions (particularly those directly targeting these nodes in tandem), as the nodes involved may help not only spread its effects to the other layers but also prevent further spread of dysfunction to nodes across network layers.

We found no GMV bridge nodes. This result is inconsistent with the multi-layer network study by Simpson-Kent and colleagues (Simpson-Kent et al., 2021), who showed that bridge brain nodes were stronger than the bridge cognitive nodes in a sample of young struggling learners. This may be due to the low between-layer network density and edge weights in our mTBI sample, particularly between the GMV layer and the other two (mental health and cognition) layers. In turn, this could be partly explained by the lack of significant differences in GMVs between mTBI and controls. In addition, future studies should investigate regions beyond those in the CEN and SN networks or use brain measures other than GMV to assess brain connectivity, such as fibre density (derived from diffusion weighted imaging) or functional connectivity (extracted from functional MRI).

### 4.3 Clinical implications

Bridge centrality measures can inform the development of treatment strategies to improve mental health and cognitive functioning in individuals with TBI. Specifically, the bridging nodes identified in our cutting-edge multilayer network analyses suggest potential targets to develop more customized, efficient, and efficacious treatment programs that target key nodes involved in the mental health symptoms or cognitive deficits of people with mTBI (Blanken et al., 2021), provided they are validated with a larger sample that yields centrality measures that are more stable (see section 4.4 Limitations and conclusions below). For example, a computerized cognitive training program targeting processing speed, such as BrainGames (Verhelst et al., 2019) or Cogmed (Caeyenberghs et al., 2016), or immediate verbal recall training (Lambez and Vakil, 2021) may lead to improved cognitive performance, which may result in improvements in mental health in persons with mTBI.

Given that insomnia severity was the node with the highest bridge strength, treatment could also focus on disturbed sleep as a modifiable treatment target as it has high likelihood of improving outcomes in mTBI, including improvement in cognitive performance (Chiu et al., 2014, Killgore et al., 2020, Sanchez et al., 2022) as well as depression, anxiety (for a review see Saravanan et al., 2024) and fatigue (Ouellet and Morin, 2007). One promising treatment could include transcranial direct current stimulation to *DMPFC*, which was recently shown in a randomized, double-blind, controlled trial to be effective in sleep promotion and daily sleepiness recovery in patients with chronic insomnia (Li et al., 2025). This finding is supported by our results, which revealed *DMPFC* was the only GMV layer node *insomnia severity* was linked to. The foregoing discussion highlights the utility of the multivariate approach implemented in this paper, as it may allow to uncover treatment pathways such as the one outlined above that can be tracked across intra and interlayer edges from the DMPFC, to the strongest bridge node (insomnia severity), through to depression and anxiety, and even potentially to cognitive nodes.

### 4.4 Limitations and conclusions

One limitation of our study is that the findings are based on a single time-point. A longitudinal analysis is required to examine changes in the multilayer networks in individuals with mTBI over time. We did not use other timepoints from the TRACK-TBI dataset (1 week and 12 months post-injury) due to the high number of missing cells for the behavioural and/or brain data of the other time points. Advances in statistical modelling will enable us to conduct longitudinal multilayer network analyses in future work, which will allow inferences about the time-related dynamics of these multilayer networks. Another limitation is that our study included mTBI participants only. Future multilayer network studies should include samples at different severity levels of TBI. Also important will be to use data from a broad spectrum of TBI populations, such as those from the ENIGMA TBI working group (Dennis et al., 2021, Keleher et al., 2023, Olsen et al., 2021).

Another shortcoming of our study is that, while both the bi- and tri-layer networks had sufficient stability, they should be interpreted with caution as their CS was below 0.5 (but above the 0.25 cutoff for interpretability). This may stem from the high degree of heterogeneity that characterizes people with mTBI. While the sample size in the present multilayer network study is considerably larger than those commonly used in standard univariate neuroimaging studies in TBI (Hellstrøm et al., 2017, Livny et al., 2017, Rostowsky and Irimia, 2021), our stability estimates suggest that even larger samples be used for this type of analysis so that node centrality estimates can be generated that are stable. The results of the present paper therefore need to be replicated and validated in a larger sample with more stable centrality measures.

We would like to underscore, furthermore, that, as noted in the methods section, the 0.5 and 0.25 cutoffs should not be taken as definite guidelines. These cutoffs were set for psychometric networks (not networks comprising psychometric and brain measures) and are based on simulations conducted with reference to single layer centrality indices (not multilayer bridge centrality indices). Also, consistent with our results, Simpson-Kent and colleagues (2021) estimated a multilayer network comprising psychological and brain layers (one of only three other such papers, according to our knowledge) and found that the CS scores were all below 0.5. Future studies are in any case needed where simulations are conducted to determine CS cutoffs with reference to multilayer data that involves psychometric and neuroimaging measures as well as bridge centrality measures.

Given that a substantial proportion of mTBI participants reported having experienced either subthreshold, moderate or severe symptoms of insomnia and that mTBI participants consequently differed from controls in this regard, future studies with large enough samples will be required to ascertain if the present network connectivity patterns are generalizable across these levels of *insomnia severity*.

Despite these limitations, to the best of our knowledge, ours is the first study to use a multilayer network approach that simultaneously models brain-behaviour relationships in individuals with mTBI. Using bridge strength centrality, we were able to identify nodes, including *insomnia severity, immediate verbal memory*, *somatization*, and *processing speed,* that work as bridges across network layers. These bridges can be considered as potential targets for intervention and can help pinpoint strategies (*e.g.*, using computerized cognitive training programs, sleep-promoting interventions) to improve outcomes in mTBI.

## Funding sources

KC is supported by a Veski Fellowship. The Victorian Near-miss Award Pilot is administered by Veski for the Victorian Health and Medical Research Workforce Project on behalf of the Victorian Government and the Association of Australian Medical Research Institutes.

## CRediT authorship contribution statement

**Juan F. Domínguez D.:** Writing – original draft, Visualization, Methodology, Formal analysis, Data curation, Conceptualization. **Mervyn Singh:** Writing – review & editing, Visualization, Methodology, Formal analysis, Data curation, Conceptualization. **Lyndon Firman-Sadler:** Writing – review & editing, Methodology. **Jade Guarnera:** Writing – review & editing. **Ivan L. Simpson-Kent:** Writing – review & editing, Methodology. **Phoebe Imms:** Writing – review & editing, Data curation. **Andrei Irimia:** Writing – review & editing, Resources, Project administration. **Karen Caeyenberghs:** Writing – original draft, Supervision, Resources, Project administration, Methodology, Conceptualization.

## Declaration of competing interest

The authors declare that they have no known competing financial interests or personal relationships that could have appeared to influence the work reported in this paper.

## Data Availability

Data will be made available on request.
